# The power of three-dimensional printing technology in functional restoration of rare maxillomandibular deformity due to genetic disorder: a case report

**DOI:** 10.1186/s13256-021-02741-5

**Published:** 2021-04-12

**Authors:** Daniel Oren, Amiel A. Dror, Tania Bramnik, Eyal Sela, Igal Granot, Samer Srouji

**Affiliations:** 1 Oral and Maxillofacial Surgery, Oral Medicine Institute, Galilee Medical Center, Nahariya, Israel; 2 Department of Otolaryngology, Head and Neck Surgery, Galilee Medical Center, Nahariya, Israel; 3 The Azrieli Faculty of Medicine, Bar-Ilan University, Safed, Israel

**Keywords:** Three-dimensional printing, Thalassemia major (beta-thalassemia major), Hereditary diseases, Bone marrow diseases, Bone demineralization, Genetic diseases

## Abstract

**Background:**

Thalassemia is an inherited autosomal recessive blood disorder causing abnormal formation of hemoglobin, known as a syndrome of anemia with microcytic erythrocytes. It is the most common genetic disorder worldwide, with a high prevalence among individuals of Mediterranean descent. The state of homozygosity of the beta-globin mutated gene is known as beta-thalassemia major, and these patients require regular blood transfusions and iron chelation therapy for survival. The rapid loss of red blood cells among affected individuals activates compensatory mechanisms of excessive medullary and extramedullary hematopoiesis, leading to severe skeletal bone deformity.

**Case presentation:**

We present the case of a 39-year-old Bedouin male, diagnosed with beta-thalassemia major at infancy, with diagnosed homozygosity for the intervening sequence 2-1 (guanine > adenine) mutation. Since early infancy, he started receiving blood transfusions with a gradual increase in treatment frequency through adulthood due to the severe clinical progression of the disease. He was referred to the oral and maxillofacial surgery department at Galilee Medical Center to evaluate his facial deformity in the upper jaw and treat his severe periodontal disease. The patient presented maxillary overgrowth, and severe dental deformity resulted in progressive disfigurement and difficulty chewing, swallowing, and speaking. To address the challenge of surgical treatment, we utilized the advantage of three-dimensional planning and printing technology to simulate the optimal result. Resection of maxillary bone overgrowth and insertion of custom-made subperiosteal implants were followed by rehabilitation of both jaws to the patients' satisfaction at 3-year follow-up.

**Conclusions:**

The ongoing implementation of state-of-the-art technologies such as virtual reality and three-dimensional printing has become a prominent component in surgical toolsets. Comprehensive case simulation and accurate planning before surgery will improve surgical results and patient satisfaction. This approach is highly advocated when approaching a case of rare maxillofacial deformity associated with either genetic or orphan diseases.

## Background

Thalassemia is an inherited autosomal recessive blood disorder that causes abnormal formation of hemoglobin. It is the most common genetic disorder worldwide, with an exceptionally high prevalence among individuals of Mediterranean descent. In thalassemia patients, the synthesized hemoglobin is mutated either in alpha or beta sequences, which causes various disabilities ranging from mild hypochromic anemia to severe, chronic, and lifelong transfusion-dependent anemia [1, 2].

**Fig. 1:**
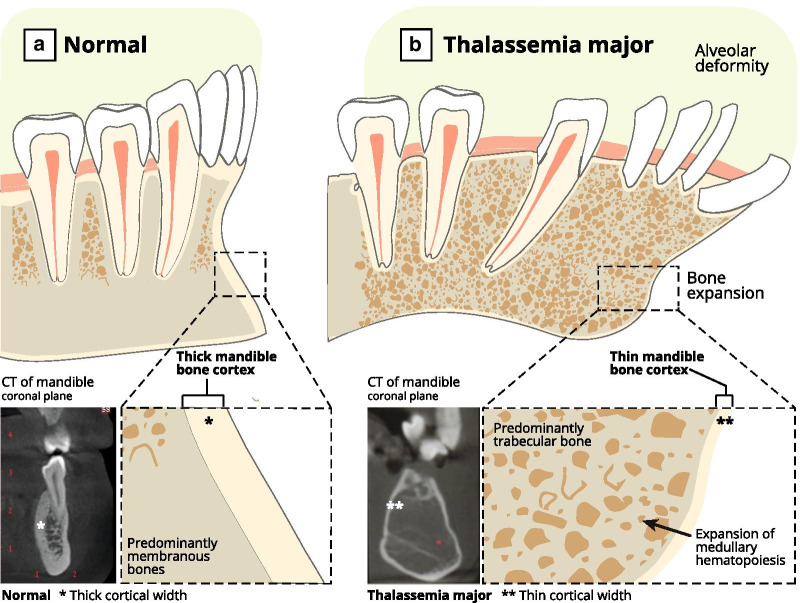
Illustration demonstrating normal mandibular bone **a** compared with bone deformity of thalassemia-major patient presenting with expanded medullary and bone marrow cavities and reduced cortex thickness leading to displacement of the dentition

To maintain hemoglobin within the normal range, blood transfusion therapy is always employed. When hemoglobin is below the standard baseline, bone marrow stimulation and expansion occur, resulting in facial deformities manifested by prominence of the cheekbones, depression of the nose, and protrusion or flaring of the maxillary anterior teeth [3, 4]. These deformities stem from marrow erythroid hyperplasia, which expands the medullary cavities and reduces the cortical structures. Marrow expansion causes dentition displacement, resulting in malocclusion, mastication, swallowing, and speech difficulties [5]. Patients with beta-thalassemia major are at high risk for dental caries, periodontal diseases, and oral infections [6]. They have smaller tooth crowns, reduced dental arch dimensions, and dental discoloration [7] (Fig. 1).

The associated bone deformities, expansion, and extensions triggered by bone marrow proliferation within beta-thalassemia patients' jaws lead to cortical bone thinning and strength reduction. As a result, primary stability for regular dental implants is absent, leading to ultimate integration failure.

Here, we describe a case of facial deformity correction in a beta-thalassemia-major patient, which heavily relied on a computer-based information system, followed by surgical procedures to resect maxillary bone overgrowth and insert custom-made implants for rehabilitation of both jaws.

## Case presentation

A 39-year-old Bedouin male, diagnosed with a beta-thalassemia major at infancy, was referred by his hematologist to the Oral and Maxillofacial Surgery Department at Galilee Medical Center for evaluation of his facial deformity in the upper jaw and to treat his severe periodontal disease. Despite his severe condition, the patient never visited any dental clinic. His parents were both asymptomatic carriers of one altered beta-globin gene, which affected our patient and his younger brother, who did not require any oral and maxillofacial intervention. He began receiving blood transfusions in early infancy, and his blood requirement increased gradually, reaching 400 mL/kg/year of packed red blood cell.

Remarkable muscle wasting as a result of severe malnutrition was noted on physical examination. The patient required the assistance of crutches to walk. He weight was 40 kg, height 170 cm, and body mass index 13. He was pale and jaundiced.

The patient presented maxillary hyperplasia with severe periodontitis, which interfered with eating, thereby affecting his social life. Complete blood count showed pancytopenia as a sign of hypersplenism, and ferritin levels were as high as 17,133 ng/mL during acute periods. Iron chelation treatment compliance was very poor. All coagulation factor levels were low, suggesting liver cirrhosis.

**Fig. 2:**
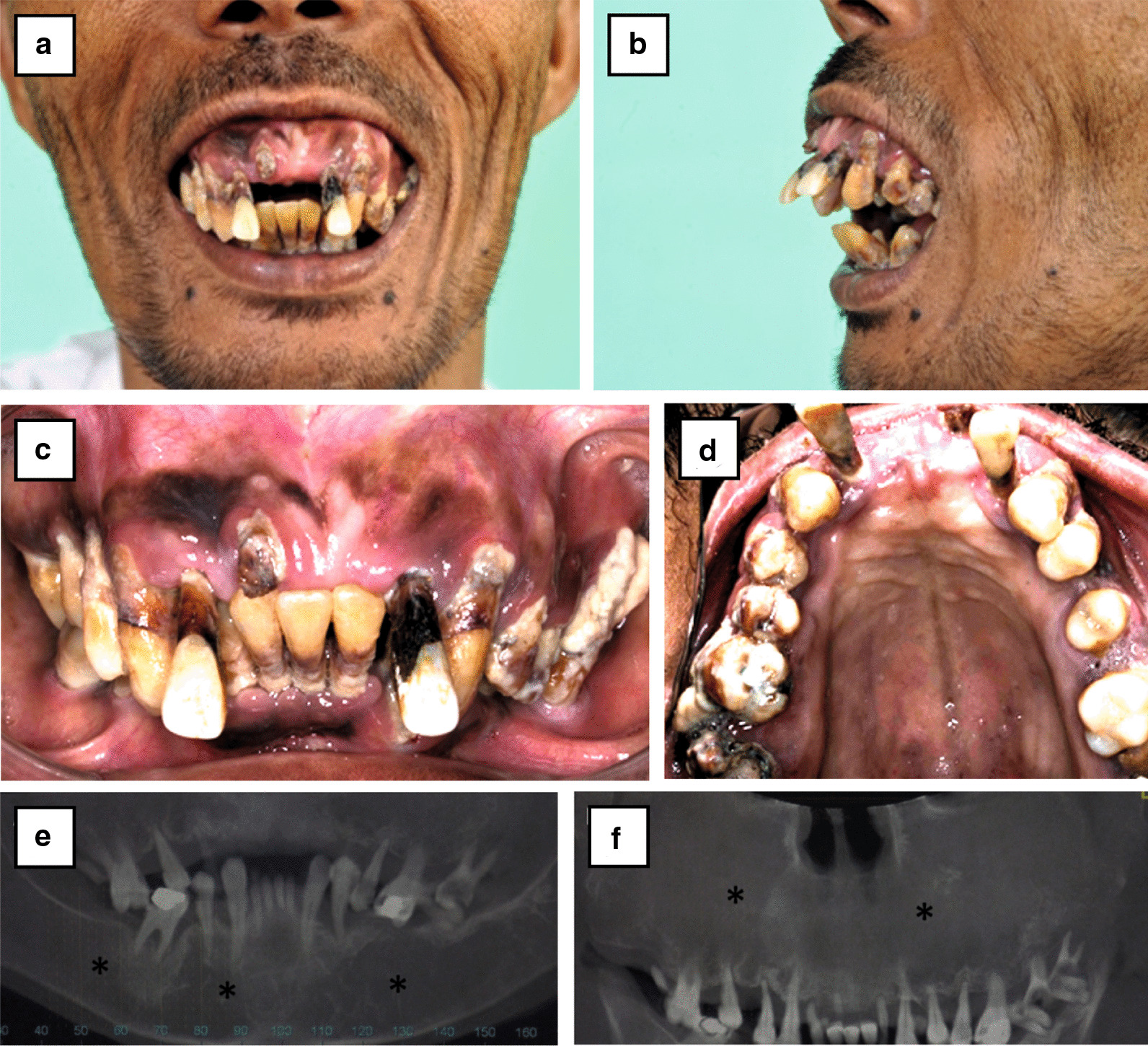
Preoperative images. **a** and **b** Frontal and lateral profile showing severe exposure of premaxilla and gummy smile. Intraoral images. **c** Occlusion. **d** Upper jaw maxilla. Dental CT. **e** panoramic lower jaw mandible. **f** Upper jaw maxilla. * Severe periodontitis, osteoporosis, osteopenia of alveolar bone, and bilateral obliteration of maxillary marked with an asterisk (*)

Maxillary overgrowth and severe dental deformity resulted in progressive disfigurement and difficulty in chewing, swallowing, and speaking. Physical examination revealed classical prominent malar bones, a saddle nose, and excessive premaxillary growth in all dimensions. Severe lip incompetence and excessive maxillary gingival display were apparent (severe gummy smile). Intraoral examination showed the extent of the vertical excess of premaxilla, and severe periodontal disease in both jaws, very poor oral hygiene, absence of teeth 21 and 11, and overeruption of teeth 22 and 12. Radiographic examination utilizing dental and 3D computed tomography (CT) showed severe periodontal disease with a floating appearance, and the jaws showed a widening of the medullary space and thinning of the mandible cortices maxilla (bowing appearance), with negligible trabecular bone. Also, the maxillary sinus was fully obliterated (Fig. 2).

**Fig. 3:**
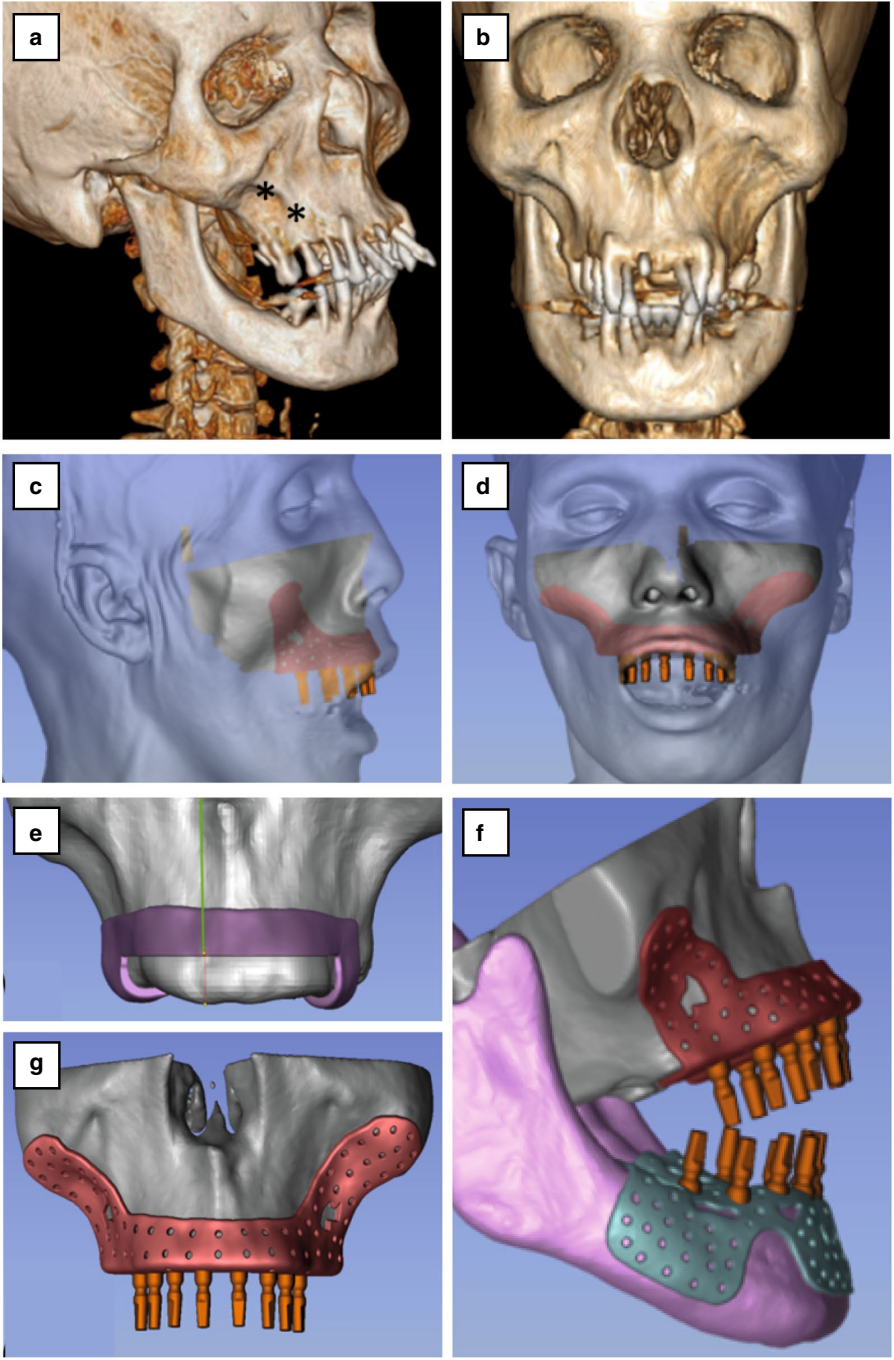
3D computed tomography scan of the patient’s skull. **a**,**b** Frontal lateral view showing prominent overgrowth of maxilla. *Periodontal (gum) disease. **c** Lateral 3D view and **d** frontal 3D view of soft tissue superimposed with hard tissue. Surgical plan. **e** Design level of osteotomy in maxilla. **f** Design of implants in maxilla and mandible with occlusion relation design of implant maxilla. **g** Design of implants in maxilla and mandible with occlusion relation and design of implant maxilla

The treatment plan considered the maxillary deformity and the quality of bone in both jaws. No endosseous implant-supported fixed prosthesis was considered because of the lack of stable cortical bone, expansion of bone marrow in both jaws, and near absence of trabecular bone. For this reason, we planned to fabricate a custom-made subperiosteal dental implant with extension to both malar bones and to the external oblique line at the mandible. The surgical plan involved extraction of all the teeth in both jaws, and resection of the vertical excess concerning the maxillary occlusal plane and the root of the zygoma. The osteotomy level considered the relation between the upper jaw and the upper lip for the future exposure of upper teeth. Virtual computerized planning of the surgery and implant angles/direction was performed with ABGuidedService software (AB Dental Devices Ltd.) (Fig. 3). The subperiosteal implants were printed from titanium Ti64 Gr5 powder by a direct metal laser sintering (DMLS) 3D printer (EOSINT M 280, EOS, Germany) utilizing the CT-scan model. A surgical wafer was fabricated for the osteotomy level. A preoperative workup included several laboratory tests, confirming average albumin level with preserved renal and hepatocellular function. Laboratory evaluation is critical in patients with a tendency for malnutrition to avoid potential complications such as delayed wound healing. Presurgical management included transfusion of two units of packed red blood cells, two units of fresh frozen plasma, 1 g tranexamic acid, and 2 g first-generation cephalosporin.

The surgery was performed under general anesthesia, utilizing nasal intubation and local anesthesia (2% lidocaine with 1:100,000 epinephrine) infiltrate in both jaws' vestibule. Gingival alveolar buccal and palatal incisions were made, and a subperiosteal flap was raised in both jaws. An additional oblique vertical releasing incision was made on both sides of the maxilla to explore the zygomatic buttress. A subperiosteal oblique releasing incision was made in both retromolar dissections at the mandible. The dissection revealed a partial lack of cortex at the maxilla and thin parts, which showed only loose honeycombed medullary bone.

Full-mouth tooth extraction was performed, and a 12-mm vertical excess was resected to ensure proper relation between future upper teeth rehabilitation and lip. Resection was made using an oscillating saw guided by a prefabricated surgical wafer in the upper and lower jaw, followed by osteotomy and alveoplasty at the extraction sites to fit the custom-made implants. After fitting the custom-made implants in both jaws, it was fixed using titanium alloy (Ti-6Al-7Nb) 2.4-mm screws (MatrixMANDIBLE, DePuy Synthes) in the zygomatic buttress, retromolar area, and chin/genio. The flaps in both jaws were then released to be sutured without tension, with vicryl 30.

**Fig. 4:**
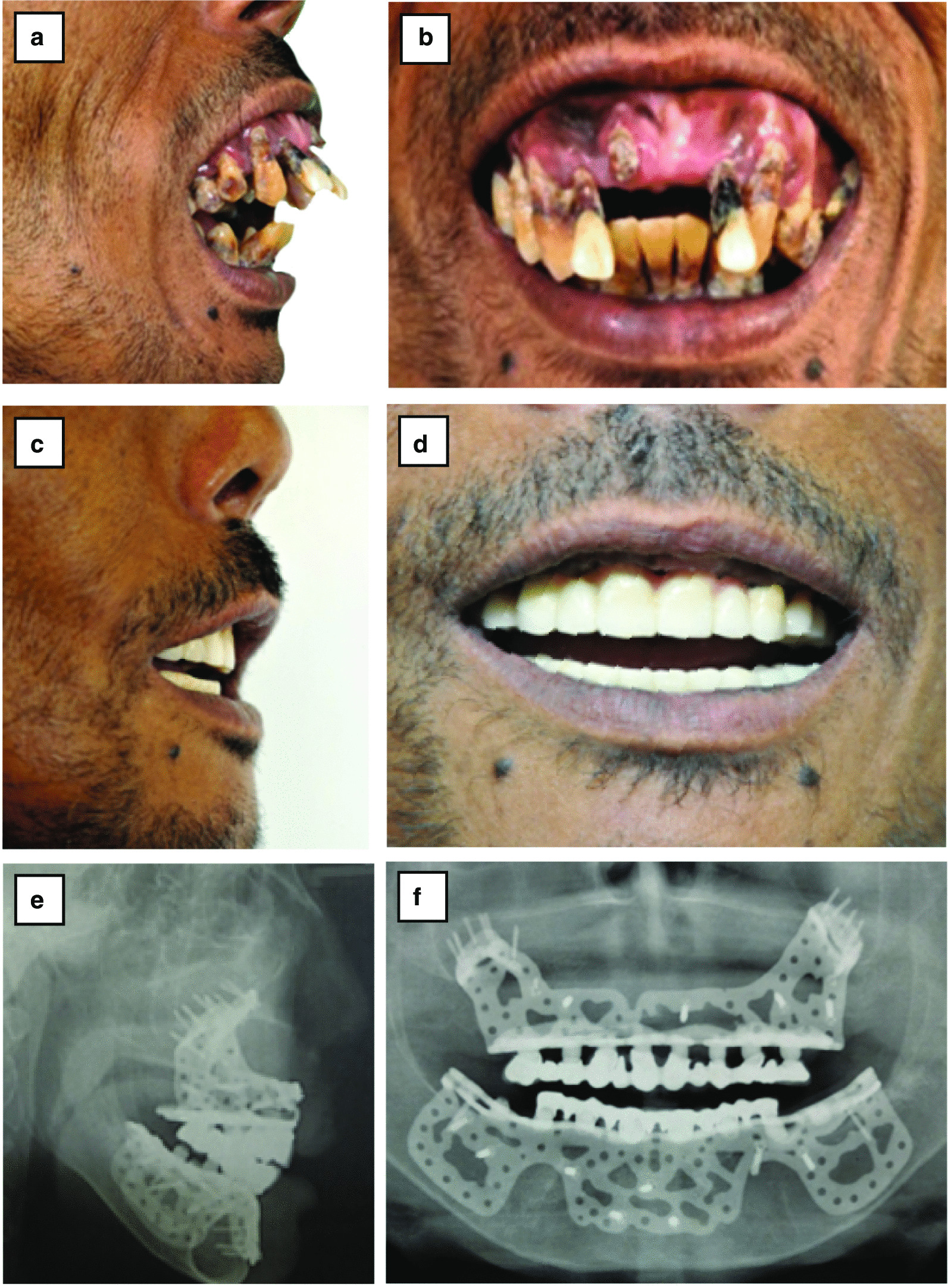
Comparison of preoperative **a** and **b** and postoperative lateral and frontal view. **c** and **d** Post-rehabilitation picture of upper and lower jaw fixed prosthesis. **e** and **f** Postoperative radiographic X-ray. **e** and **f** Panoramic lateral cephalometric view X-rays

The patient lost approximately 650 mL blood, mostly attributed to persistent oozing from the hyperplastic marrow. The patient received 1 U packed red blood cells intraoperatively. His immediate postoperative hemoglobin reading was 9.8 g/dL, and 3-month serial complete blood counts showed he maintained hemoglobin levels. Overall, the patient tolerated the procedure well and encountered no postoperative complications. The patient received IV antibiotics and steroids during hospitalization and was released from the hospital after 6 days. Postoperative panoramic and lateral cephalometric X-rays showed good fitting of the implant in both jaws (Fig. 4).

Oral mucosa healing above the implant was good and free of infection and dehiscence of the gingiva. A dental impression was taken for the upper and lower fixed prosthesis at the 1-month follow-up, inserted to restore the patient occlusion 6 weeks after surgery. A final dental X-ray examination was performed after both jaws with the fixed prosthesis had been rehabilitated.

**Fig. 5:**
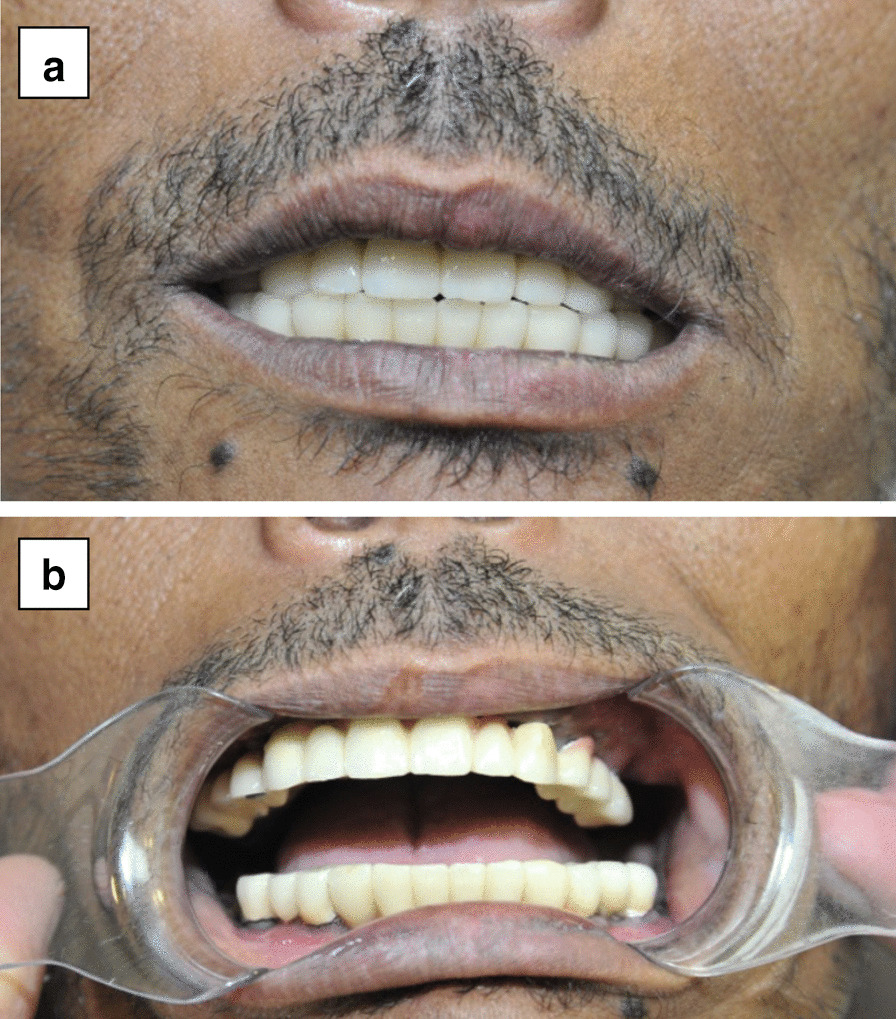
Three years postoperative images. **a** and **b** Upper and lower jaw fixed prosthesis

In the 3-year follow-up visits conducted after the operation, the patient reported a significant improvement in facial profile and no longer exhibited a gummy smile. The substantial improvement in lip closure (competent lips) and stable jaw occlusion led to significant functional improvements. The patient reported a significant improvement in his chewing capability, allowing him to eat and swallow solid foods and significantly improve his pronunciation and speaking (Fig. 5). No significant soft tissue dehiscence was observed in the follow-up period.

## Discussion

Beta-thalassemia is a hereditary disorder characterized by a deficiency in the synthesis of beta-globin chains, a constituent of hemoglobin responsible for carrying oxygen within red blood cells. Patients with this disease suffer from skeletal deformity, particularly in the skull and maxilla, stemming from marrow erythroid hyperplasia that expands to medullary cavities, culminating in the attenuation of the cortical structures.

The clinical management of beta-thalassemia is based on adequate, safe, and chronic blood transfusion therapy to prevent iron overload. Maintaining the hemoglobin level at 9–10 g/dL improves patients' wellbeing while simultaneously suppressing enhanced erythropoiesis. Beta-thalassemia patients can develop iron overload that may impair vital organs, that is, the heart and lungs. Impairment in cardiac and pulmonary function is the leading cause of death and morbidity in thalassemia patients. As such, laboratory analyses are performed routinely. These include complete blood counts, serum chemistries, liver function tests, coagulation profile, iron panel, and urinalysis, as well as a thorough evaluation of chest radiography and electrocardiography.

While not an ultimate cure for thalassemia patients, treatment of anemia and endogenous erythropoiesis suppression prevents extramedullary hematopoiesis and skeletal changes; nevertheless, despite treatment, most patients with thalassemia major do develop facial deformities of varying extent. However, only a few cases have been reported in the literature [8–10]. Our consensus plan was to extract all teeth and simultaneously repair and excise overgrowth protrusions in the maxilla due to the skeletal changes in the presented case. The challenge we faced was to rehabilitate and restore balanced occlusion of the maxilla with the mandible. The option of installing individual standard dental implants (that is, not embedded together within the arch) was impossible in this case because of trabecular bone deformity associated with progressive thalassemia major disease. If installed, standard implants would have no primary stability, and it was doubtful that implant osseointegration would be successful.

Subperiosteal implants were introduced in the early 1940s and are placed subperiosteally between the periosteum and the residual alveolar bone [11–13]. While subperiosteal implants gained some popularity, they were subsequently replaced by endosseous implants, as proposed by Branemark [14]. Initially, the fabrication technique of the subperiosteal implants was complex and led to patient discomfort since it required physical impression of the residual bone anatomy and fabrication of the metal framework in the laboratory, which resulted in less-than-ideal fit and eventually led to a higher risk of postoperative infections and complications [13, 15–17].

In the last few years, the digital revolution of data acquisition, computer-assisted-design/computer-assisted-manufacturing (CAD/CAM) software [18], and DMLS techniques [19] have allowed the fabrication of highly accurate custom-made subperiosteal implants. These implants have become useful in cases of severe bone atrophy, which does not allow for the placement of endosseous dental implants [20].

To the best of our knowledge, this is the first description of a surgical resection repairing maxillary overgrowth and simultaneously inserting a custom-made subperiosteal dental implant for the rehabilitation of both jaws in a patient with a thalassemia-induced facial deformity.

## Conclusions

Treatment of patients with thalassemia major may lead to other beneficial surgical interventions that will be appropriate for patients with thalassemia and patients with other deformities of maxillary excesses, mandibular deficiency, or even traumatic physical injuries. Our report highlights the implication of 3D planning in the treatment plan of complex clinical manifestations of genetic disease. Early simulation and accurate planning will benefit patients with rare deformities by providing a sustained and successful surgical solution.

## Data Availability

All data generated or analyzed during this study are included in this published article.

## Abbreviations

3D: Three-dimensional
CT: Computed tomography
